# Nickel exposure reduces enterobactin production in *Escherichia coli*

**DOI:** 10.1002/mbo3.691

**Published:** 2018-07-30

**Authors:** Clorissa L. Washington – Hughes, Geoffrey T. Ford, Alsten D. Jones, Kimberly McRae, F. Wayne Outten

**Affiliations:** ^1^ Department of Chemistry and Biochemistry University of South Carolina Columbia South Carolina

**Keywords:** enterobactin, *Escherichia coli*, iron, nickel, siderophore

## Abstract

*Escherichia coli* is a well‐studied bacterium that can be found in many niches, such as industrial wastewater, where the concentration of nickel can rise to low‐millimolar levels. Recent studies show that nickel exposure can repress pyochelin or induce pyoverdine siderophore production in *Pseudomonas aueroginosa*. Understanding the molecular cross‐talk between siderophore production, metal homeostasis, and metal toxicity in microorganisms is critical for designing bioremediation strategies for metal‐contaminated sites. Here, we show that high‐nickel exposure prolongs lag phase duration as a result of low‐intracellular iron levels in *E. coli*. Although *E. coli* cells respond to low‐intracellular iron during nickel stress by maintaining high expression of iron uptake systems such as *fepA*, the demand for iron is not met due to a lack of siderophores in the extracellular medium during nickel stress. Taken together, these results indicate that nickel inhibits iron accumulation in *E. coli* by reducing the presence of enterobactin in the extracellular medium.

## 1 INTRODUCTION

Siderophores are small molecules with high affinity for ferric iron and are produced by bacteria under iron‐limiting conditions (Matthew, Jenul, Carlier, & Eberl, 2016; Neilands, 1995). Siderophores are exported from the cell into the extracellular environment to bind and transport ferric iron (Fe^3+^) back into the cell. Siderophores have also been shown to promote intracellular iron accumulation in *Pseudomonas aeruginosa* (Braud, Geoffroy, Hoegy, Mislin, Schalk, 2010), alter metal toxicity in uropathogenic bacteria (Chaturvedi, Hung, Crowley, Stapleton, & Henderson, 2012), protect against oxidative stress (Adler et al., 2014), and detoxify metal‐contaminated soils (Nair, 2007).

Enterobactin is the predominant siderophore secreted by *E. coli* under iron‐limiting conditions. Niches where those conditions exist include soil (Solomon, Yaron, & Matthews, 2002), plants (Itoh et al., 1998), and mammalian intestinal systems or pathogenesis sites (Van Elsas, Semenov, Costa, & Trevors, 2011). In *E. coli,* enterobactin is synthesized by the concerted action of the gene products encoded by the *entCEBA* operon (Crosa & Walsh, 2002; Ma & Payne, 2012; Walsh, Liu, Rusnak, & Sakaitani, 1990). It is then exported from the cytoplasm by the inner membrane transporter protein EntS (Bleuel et al., 2005; Furrer, Sanders, Hook‐Barnard, & McIntosh, 2002; Miethke & Marahiel, 2007). Extracellular ferric iron binds to apo‐enterobactin forming Fe^3+^–enterobactin. Fe^3+^–enterobactin is preferentially imported back into the cell by binding to the outer membrane transporter protein FepA, then to FepB in the periplasm, and through the FepCDG transporter in the inner membrane (Braun, 1995; Larsen, Foster‐Hartnett, McIntosh, & Postle, 1997). Enterobactin is the cyclized form of three N‐(2, 3‐dihydroxybenzoyl)‐L‐serine monomeric units (Ehmann, Shaw‐Reid, Losey, & Walsh, 2000; Gehring, Bradley, & Walsh, 1997; Gehring, Mori, & Walsh, 1998 *;* Shaw‐Reid et al., 1999). The backbone of intracellular Fe^3+^–enterobactin must be hydrolyzed by the esterase Fes and the iron reduced by YqjH (NfeF), releasing ferrous iron from the tightly chelated ferric complex (Brickman & McIntosh, 1992; Bryce & Brot, 1972; Greenwood & Luke, 1978; Langman, Young, Frost, Rosenberg, & Gibson, 1972; Miethke, Hou, & Marahiel, 2011). The cleavage of intracellular enterobactin (cyclo‐tris(2,3‐dihydroxy‐N‐benzoylseryl) by Fes results in the production of four linear hydrolysis products including: the nonhydrolytically cleaved trimer (N, N’, N’’‐tris(2,3‐dihydroxybenzoyl)‐O‐(a‐aminoacrylyl)‐O‐seryl serine), the hydrolytically cleaved trimer (N, N’, N’’’‐tris(2,3‐dihydroxybenzoyl)‐O‐seryl‐O‐seryl serine), a linear dimer (N, N’‐bis(2,3‐dihydroxybenzoyl)‐O‐(a‐aminoacrylyl)‐O‐seryl serine), and a linear monomer (2,3‐dihydroxy‐N‐benzoylserine) (Lin, Fischbach, Liu, & Walsh, 2005; Brickman & McIntosh, 1992; O'Brien & Gibson, 1970).

Siderophores like enterobactin have been shown to impact homeostasis of other essential or toxic metals. For example, enterobactin can facilitate the reduction in Cu^2+^ to Cu^1+^ and increase copper cytotoxicity in uropathogenic *E. coli* (Chaturvedi et al., 2012). Siderophores have been shown to protect against metal toxicity perhaps by chelating other metals (Chen, Jurkewitch, Bar‐Ness, & Hadar, 1994; Koh et al., 2015). The presence of the *Pseudomonas aeruginosa* siderophores pyochelin (PCH) and pyoverdine (PVD) have been shown to decrease intracellular nickel accumulation (Braud, Hannauer, Mislin, & Schalk, 2009; Braud, Hoegy, Jezequel, Lebeau, & Schalk, 2009; Braud et al., 2010). In addition, media supplementation with PCH and PVD reduced nickel toxicity in iron‐limited and iron‐supplemented media (Braud et al., 2010). However, exposure to other toxic metals can also alter siderophore expression. Siderophore production is repressed by excess molybdenum in *Azobacter vinelandii* (Duhme, Hider, Naldrett, & Pau, 1998) but aluminum increases hydroxamate siderophore production in *Bacillus megaterium* (Hu & Boyer, 1996). Copper and nickel can increase siderophore production in the presence of iron levels that are not limiting for growth in *Pseudomonas aeruginosa* (Braud et al., 2010; Visca et al., 1992). In *P. aeruginosa,* nickel has been shown to repress PCH synthesis (Visca et al., 1992) but induce PVD synthesis (Braud, Hannauer, Mislin, & Schalk, 2009) under iron‐limited conditions. Therefore, understanding the molecular cross‐talk between siderophore production, metal homeostasis, and metal toxicity in microorganisms is critical for designing bioremediation strategies for metal‐contaminated sites (Dixit et al., 2015). For example, Actinobacteria can be used to detoxify metal‐contaminated sites and break‐down complex organic matter (Albarracín, Amoroso, & Abate, 2005; Alvarez et al., 2017; Kieser, Bibb, Buttner, Chater, & Hopwood, 2000; Polti, Amoroso, & Abate, 2011; Polti, Aparicio, Benimeli, & Amoroso, 2014; Polti, Atjian, Amoroso, & Abate, 2011; Polti, García, Amoroso, & Abate, 2009). Its robust metabolic profile and the emergence of new actinobacteria species (Kaewkla & Franco, 2013) has made this class of organism a prime system for understanding the molecular details of siderophore production (Wang, Qiu, Tan, & Cao, 2014).

Recently, nickel has been shown to lower intracellular iron concentrations possibly by transcriptional repression of iron acquisition genes in *E. coli* (Gault, Effantin, & Rodrigue, 2016). Furthermore, strains lacking the stress‐responsive iron cofactor biogenesis system Suf are more sensitive to nickel stress than wild‐type *E. coli* (Wang, Wu, & Outten, 2011). Our goal in this work is to test if nickel stress alters siderophore production under iron‐limiting conditions in *E. coli*.

## 2 EXPERIMENTAL PROCEDURES

### 2.1 Bacterial strains and culture conditions

Strains used in this study are derivatives of the parent wild‐type strain *E. coli* MG1655. An individual colony was transferred from fresh Lennox broth (LB) agar plates into a 4 ml volume of LB and grown for 4‐5 hr at 37°C with shaking at 200 rpm. Cells from this culture were pelleted and washed twice in sterile 1X M9 minimal media salts; then the OD_600 nm_ was normalized to 1.0. Normalized cells were diluted 1:200 into M9 glucose minimal media containing 1X M9 minimal salts (BD Difco), 0.2% (w/v) glucose (Acros Organics), 0.2% (w/v) magnesium chloride, 0.1 mM calcium sulfate, and 0.5 g/ml Thiamine HCl (Sigma‐Aldrich). Prepared M9 media typically contained ~300 nM iron and ~70 nM nickel as measured by ICP‐MS. Cultures were incubated overnight for 18‐20 hr, at 37°C and 200 rpm, then washed and pelleted twice in sterile 1X M9 salts as described above. The resulting cell suspensions were normalized to an OD_600_ of 2.0 and diluted 1:50 into M9 gluconate minimal media with 0.2% (w/v) potassium gluconate (Alfa Aesar) to give an initial OD_600_ of 0.04. Nickel chloride (Sigma‐Aldrich) was added to describe final concentrations in the M9 gluconate minimal media, from 0 μM up to 50 μM.

Cell growth was monitored as optical density at 600 nm (OD_600_) and plotted versus time (in hours). Lag phase duration was determined using the online fitting program, DMFit (www.ifr.ac.uk/safety/DMfit), applying the no‐asymptote fitted model and parameters (Baranyi & Roberts, 1994). Stationary phase OD_600_ measurements were omitted for best fit of the model. Doubling time of the cells during the exponential phase of growth, where the steepest linear fit line could be applied, was determined using the Online Doubling Calculator (http://www.doubling-time.com/compute.php) (Roth, 2006).

### 2.2 Inductively – coupled Plasma Mass Spectrometry (ICP‐MS)

Preparatory cell growth in LB and glucose minimal media was conducted as described above**.** Cell cultures were then grown in 2 L M9 gluconate minimal media with or without 50 μM nickel chloride in a 4 L culture flask at 37°C and 200 rpm. A total of 150 ml samples were centrifuged at 3,000*g* for 20 min and then pelleted three times at 16,000*g* with intermediate washing in 1 ml sterile, ice‐cold wash solution consisting of 50 mM EDTA tetrasodium salt, 100 mM oxalic acid, 100 mM NaCl, and 10 mM KCl, to remove any cell surface‐associated metal ions. Washed cell pellets were resuspended in a 1 ml volume of ice‐cold, sterile 1X M9 salts. A small portion of each sample was then diluted 40‐fold to record the final OD_600_. Cell resuspensions were transferred to an acid‐washed, Perfluoroalkoxy (PFA) microcentrifuge tube (Savillex Corporation) and centrifuged at 16,000*g*. After centrifugation, the supernatant was discarded and the cell pellets were frozen in liquid nitrogen. Cell pellets were stored at −80°C until ready for digestion and ICP‐MS analysis.

Samples for ICP‐MS were thawed for 15 min on ice followed by drying at 80°C for 30 min. A 400 μL volume of trace‐metal grade HNO_3_ (distilled on site at the Center for Elemental Mass Spectrometry (CEMS), University of South Carolina) was added to each sample tube and digested at 80°C for 4 hr. After digestion, each sample tube was centrifuged for 1 min at 16,000*g* and the supernatant was diluted 1:20 into MQ H_2_O, giving a final acid matrix of 3.5%. Blanks consisting of 3.5% trace‐metal grade HNO_3_ only in MQ H_2_O (18MΩ) were made and prepared in the same manner as the cell samples. Standard element solutions (Inorganic Ventures) were also prepared in the same final acid matrix of 3.5% to establish a limit of detection and a calibration curve for determining the concentrations of each metal analyzed. The isotopes of biologically relevant transition metals with masses of ^56^Fe, ^58^Ni, ^64^Zn, ^55^Mn, and ^63^Cu were selected for analysis based on natural abundances. Samples were analyzed under medium resolution to resolve polyatomic interferences (e.g. ^40^Ar^16^O for ^56^Fe) on a Thermo Element 2 High Resolution ICP‐MS instrument operated by CEMS at the University of South Carolina. A cyclonic spray chamber (Elemental Scientific) was used for delivery of sample into the instrument.

### 2.3 β‐Galactosidase assays for promoter‐lacZ fusion strains

Wild‐type *E. coli* MG1655 strains containing Φ*fepAp‐LacZ* (PK9849), Φ*iscRp‐lacZ* (PK7571), and Φ*sufAp‐lacZ* (PK7722) were kindly provided by Patricia Kiley (University of Wisconsin – Madison) (Giel, Rodionov, Liu, Blattner, & Kiley, 2006). All cells were initially plated on LB with 30 μg/ml kanamycin overnight at 37°C. One colony was transferred to M9 glucose minimal media for approximately 18 hr at 37°C at 200 rpm. The cell culture was then diluted 1:50 to a final OD_600_ of 0.04 in 100 ml of M9 gluconate minimal media with or without 50 μM NiCl_2_ and grown for 5 hr at 37°C at 200 rpm. At various time points, cells were collected by centrifugation at 3,000*g* and resuspended in Z‐buffer (0.06 M sodium diphosphate, 0.04 M monosodium phosphate, 0.01 M potassium chloride, 0.001 M magnesium sulfate, and 0.05 M β‐mercaptoethanol). β‐galactosidase activity was measured after addition of 200 μl of 4 mg/ml ortho‐Nitrophenyl‐ß‐galactoside per ml of cells permeabilized with chloroform and SDS according to published protocols (Miller, 1972). β‐galactosidase activity was calculated and reported in Miller Units; see Equation (1) below where *t *= time of reaction and υ = volume of cells added in ml. Absorbance at 420 nm, 550 nm, and 600 nm were measured using a Beckman‐Coulter DU800 UV‐Vis Spectrophotometer. Miller units normalize β ‐galactosidase activity to total cell number via optical density at 600 nm (OD_600_) measurement.

(1) $$\mathrm{Miller}\;\mathrm{Unit}\;=1000\;*\left[{\mathrm{Abs}}_{420}-\left(1.75\;*\;{\mathrm{Abs}}_{550}\right)\right]/[t*\upsilon *{\mathrm{Abs}}_{600}$$

### 2.4 Arnow assay for catechol determination

Methods from Arnow and Ma were adapted for the quantitation of catecholate siderophore production (to include any enterobactin breakdown products) by *E. coli* under nickel stress (Arnow, 1937; Ma & Payne, 2012). Wild‐type MG1655 and Δ*fepA* strains were cultured as described above. Cells were cultured in 0.2% gluconate M9 minimal media with or without 50 μM nickel chloride. Every 2 hr 1 ml was collected from each growth, the OD at 650 nm was measured and recorded, and then each volume was cleared of cells via centrifugation at 16,000*g* for 1 min. A 500 μl volume of cleared supernatant was transferred to a clean, 4.0 ml polypropylene cuvette. A total of 500 μl 0.5N HCl, 500 μl of a 10% sodium nitrate/10% sodium molybdate mixture (Sigma‐Aldrich), and 500 μl 1N NaOH were added to the cuvette. All assay samples were measured against a blank mixture of fresh gluconate M9 minimal media with the above reagents listed for the assay. The absorbance at 515 nm was measured and recorded immediately after mixing. Arnow units were calculated using Equation (1)

(2) $$\text{Arnow Unit}=1000*[{\text{Abs}}_{515}/{\text{Abs}}_{650}]$$

### 2.5 Enterobactin purification and quantitation using FPLC

*Escherichia coli* MG1655 wild‐type and ∆*fepA* were plated onto LB and incubated overnight at 37°C. A single colony was cultured according as described above. Cultured cells were washed, normalized, and diluted to a final optical density of 0.04 in M9 gluconate minimal media, with or without 50 μM nickel chloride. Cultures were incubated for 2 hr at 37°C at 200 rpm. The cells were centrifuged for 20 min at 4°C and 8,000*g*. The supernatant was sterile filtered twice using a fresh 0.22 μm filter (Millipore) each time and a total of 1 L spent media was collected. Enterobactin and its hydrolysis products were purified using a modified form of a previously published protocol (O'Brien & Gibson, 1970). Briefly, the sterile, filtered supernatant was loaded onto a DEAE‐Sepharose Fast Flow column equilibrated with 10 mM sodium phosphate buffer, pH 7.0. 5 ml fractions were collected by eluting at 4°C using a step gradient of 0.0 M, 0.05 M, 0.15 M, 1.0 M, and 2.0 M ammonium chloride. Enterobactin and its hydrolysis products were identified based on the concentration of ammonium chloride at which they eluted and further confirmed by ESI‐MS (data not shown).

## 3 RESULTS

### 3.1 Wild‐type *E. coli* cells are sensitive to nickel stress during lag phase

**Figure 1:**
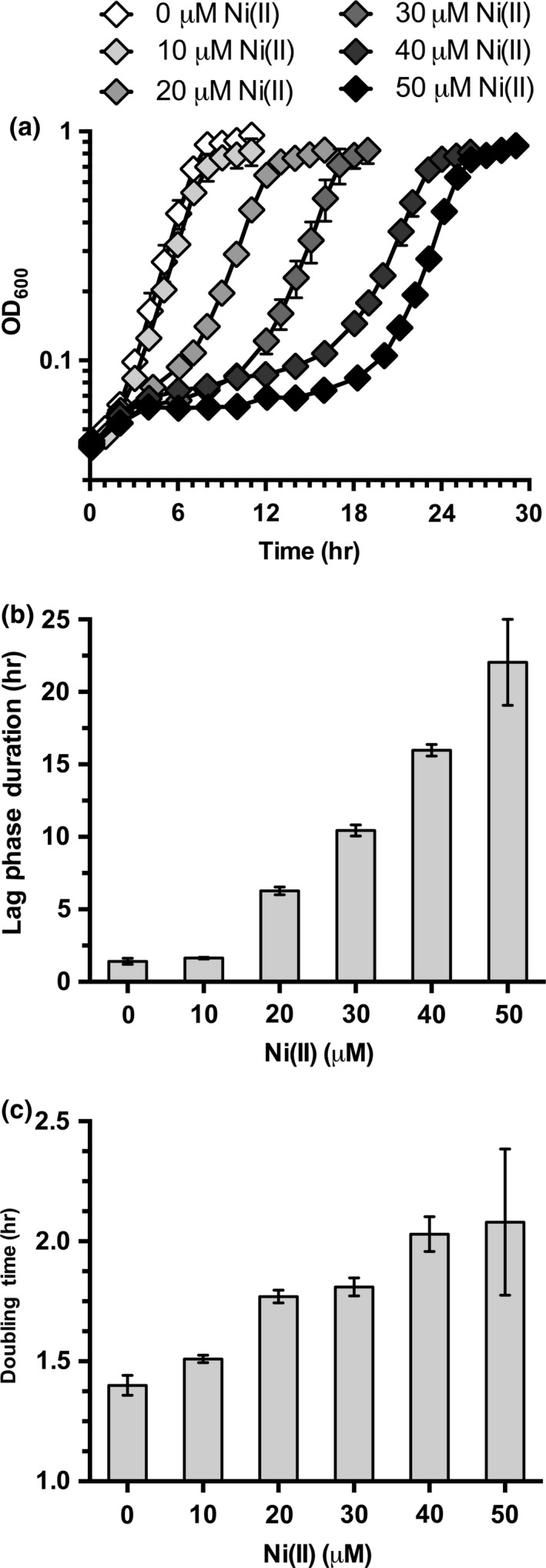
Nickel exposure extends lag phase duration. (a) Growth curves of wild type MG1655 *Escherichia coli* cells in M9 gluconate minimal media exposed to 0 μM, 10 μM, 20 μM, 30 μM, 40 μM, or 50 μM nickel chloride. (b) Lag phase duration calculated from the growth curve data shown in (a). (c) Doubling times calculated from the growth curve data shown in (a). All growths were repeated in triplicate (*n* = 3) and error bars indicate one standard deviation from the mean value

*Escherichia coli* cells have three primary stages of growth: lag phase, exponential phase, and stationary phase (Wade, 1952). Nickel is toxic at low micromolar levels (8 μM) to bacterial cells in exponential phase and nickel was shown to disrupt the zinc‐dependent metalloenzyme Class 2 Fructose‐bisphosphate aldolase, FbaA (Macomber, Elsey, & Hausinger, 2011). Nickel exposure in exponential phase *E. coli* was also shown to induce DNA relaxation and damage, possibly by inhibiting DNA replication and RecBCD‐mediated DNA repair rather than by generating reactive oxygen species (Gault et al., 2016 and Kumar, Mishra, Kaur, & Dutta, 2017). However, Rolfe et al., (2012) have shown that bacterial cells accumulate essential trace metals during *lag phase* in preparation for the transition into exponential phase. Iron uptake genes are upregulated and intracellular iron levels are increased during lag phase. During exponential phase, these genes are downregulated and intracellular iron levels decrease as the iron is progressively divided among daughter cells. To determine the effects of nickel during lag phase of *E. coli*, growth was monitored after exposure of freshly diluted lag phase cells to 0–50 μM nickel chloride (Figure 1a). Lag phase duration (Figure 1b) was quantitated using the Baranyi and Roberts model (Baranyi & Roberts, 1994) while the exponential phase (doubling time) duration (Figure 1c) was quantitated using a formula developed by Roth (2006). Lag phase duration is not significantly affected at the lower nickel toxicity range (below 10 μM). However, as the concentration of nickel increases above 10 μM, lag phase duration also increases. The doubling time does not significantly change when the nickel‐treated cells exit lag phase and enter exponential phase. Similarly, the final cell density reached in stationary phase also does not significantly change after nickel exposure in lag phase (Figure 1a).

### 3.2 Intracellular iron levels are lower in nickel‐treated *E. coli* cells

**Figure 2:**
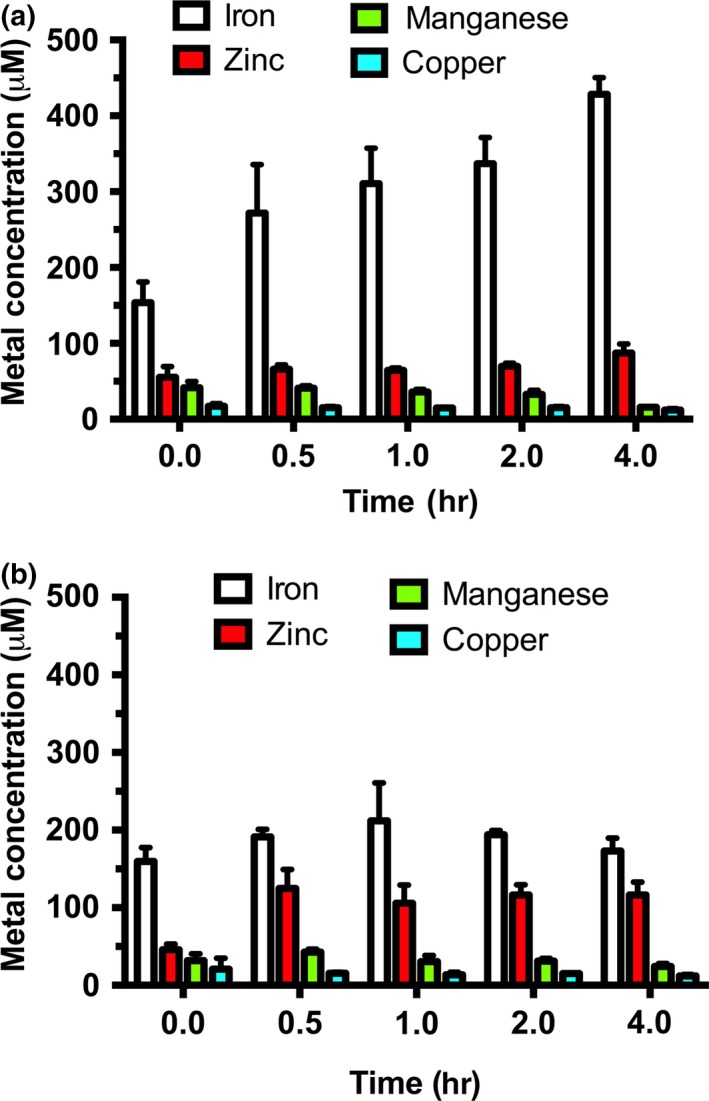
Intracellular iron levels are decreased upon exposure to nickel during the lag phase. (a) Intracellular metal concentrations were measured in wild type MG1655 *Escherichia coli* cells that were exposed to 0 μM nickel chloride using ICP‐MS. (b) Intracellular metal concentrations were measured in wild type MG1655 *E. coli* cells that was exposed to 50 μM nickel chloride using ICP‐MS. All measurements were repeated in triplicate (*n* = 3) and error bars indicate one standard deviation from the mean value

Soft metals have been shown to disrupt iron metabolism in *E. coli* (Macomber & Imlay, 2009; Ranquet, Ollagnier‐de‐Choudens, Loiseau, Barras, & Fontecave, 2007; Xu & Imlay, 2012). Recent studies link nickel stress to disruption of iron metabolism in exponential phase, possibly by inducing Fur‐dependent repression of iron uptake systems (Gault et al., 2016). To better understand the phenotypic effects of nickel toxicity, intracellular metal concentrations during lag phase nickel stress were measured using inductively coupled plasma mass spectrometry (ICP‐MS). Cells that were not exposed to nickel showed an increasing amount of iron over time during lag phase (Figure 2a). In contrast, cells exposed to a toxic level of nickel (50 μM) showed no increase in intracellular iron throughout lag phase but did accumulate nickel (Figure 2b and S1). Manganese and copper levels were not significantly affected but zinc levels were elevated in response to nickel. The increased zinc accumulation in response to nickel is consistent with disruption of zinc metalloproteins by nickel as previously reported (Macomber & Hausinger, 2011; Macomber et al., 2011).

### 3.3 High nickel exposure triggers an ‘iron starvation’ response in lag phase *E. coli* cells

**Figure 3:**
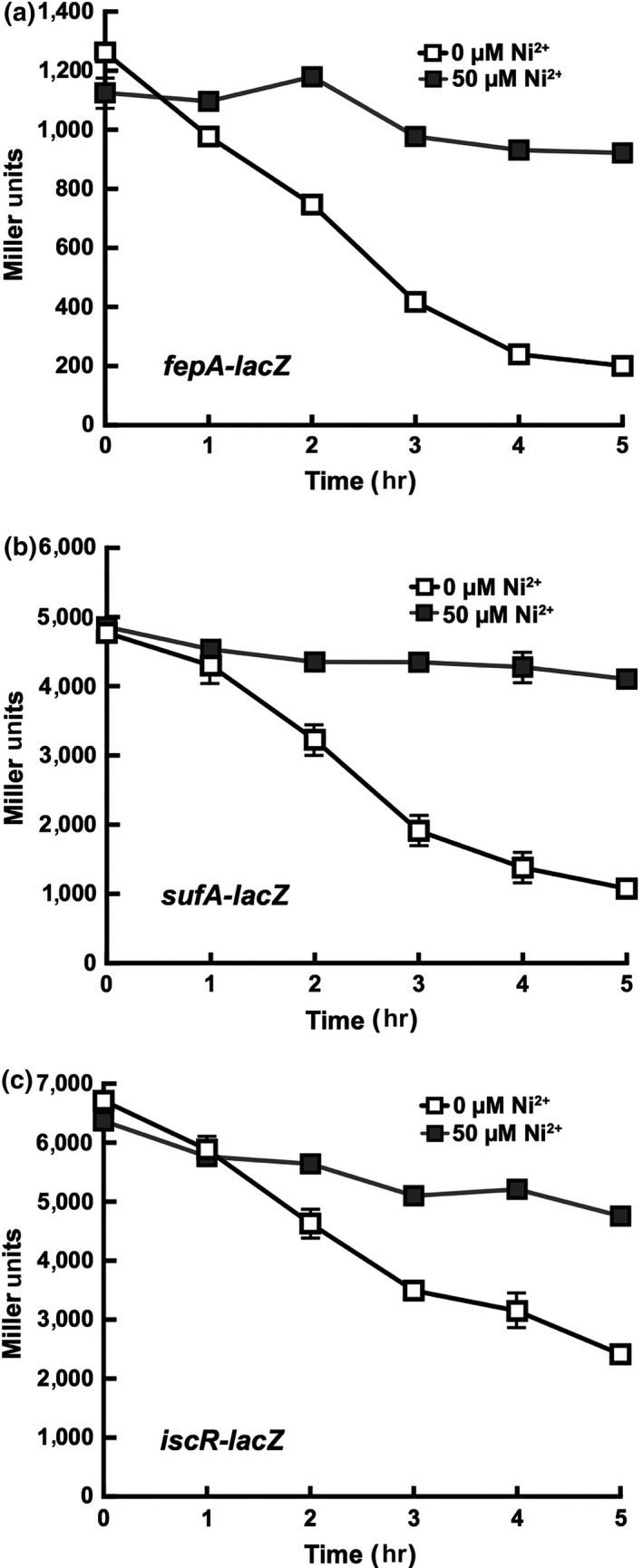
Nickel induces the Fur and IscR regulons. Relative gene expression levels are shown in Miller Units which accounts for any observed differences in bacterial cell growth or optical density at 600 nm. (a) *fepA‐lacZ* gene expression levels. (b) *sufA‐lacZ* gene expression levels. (c) *iscR‐lacZ* gene expression levels. All measurements were repeated in triplicate (*n* = 3) and error bars indicate one standard deviation from the mean value

To determine the effects of nickel exposure on intracellular iron homeostasis, expression of genes involved in iron homeostasis were monitored using a series of promoter‐*lacZ* fusion constructs in vivo. Iron uptake (*fepA*) and iron‐sulfur (Fe‐S) cluster biogenesis (*iscR, sufA*) genes are upregulated during lag phase and their expression progressively declines over time in exponential phase (Rolfe et al., 2012). The ferric uptake regulator, Fur, regulates iron homeostasis by repressing the transcription of iron uptake genes under iron replete conditions (Escolar, Perez‐Martin, & de Lorenzo, 1998; Hunt, Pettis, & McIntosh, 1994). However, when cellular demand for iron is high, Fur repression of genes like *fepA* and *sufA* that are involved in adaptation to iron starvation is reversed leading to their induction. Under normal growth conditions (in the absence of nickel), we observed that *fepA* and *sufA* gene expression levels gradually decreased over time throughout lag phase (Figure 3a,b). In contrast, nickel exposure (50 μM) results in a consistently high level of *fepA* and *sufA* expression throughout lag phase when compared to cells that were not exposed to nickel (Figure 3a,b). The nickel‐dependent upregulation of *fepA* in lag phase was also independently confirmed by RT–qPCR (Figure S2). The expression of *iscR* is auto‐regulated in response to demand for Fe‐S cluster biogenesis and is influenced indirectly by intracellular iron availability. Therefore, *iscR* expression can also be used as an indicator of intracellular iron status. Similar to that observed for *fepA* and *sufA*,* iscR* expression gradually decreased throughout lag phase in control cells but remained high throughout lag phase in nickel‐treated cells (Figure 3c). These results are consistent with other studies and clearly support the hypothesis that nickel disrupts iron metabolism in multiple growth phases in *E. coli* (Gault et al., 2016).

### 3.4 The presence of siderophores decreases in nickel‐treated *E. coli* cells

**Figure 4:**
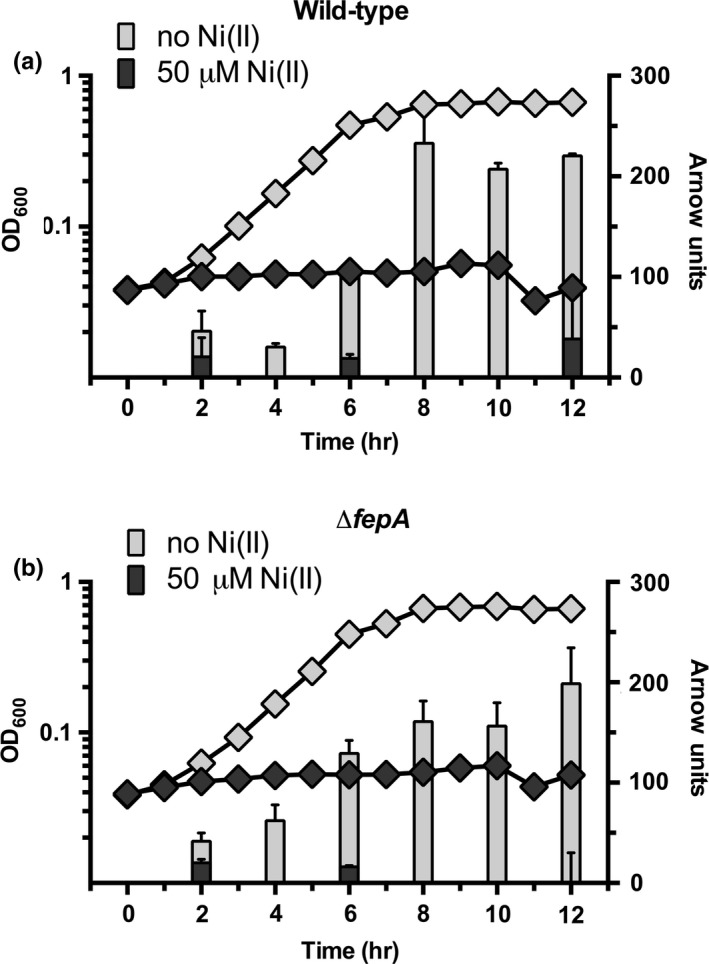
Nickel decreases levels of extracellular catecholate siderophores during lag phase. Total catecholate production is expressed in Arnow units (right axis, bars). Relative growth is expressed by optical density at 600 nm (left axis, diamonds). (a) Wild‐type and (b) Δ*fepA* MG1655 *E. coli* cells measurements are overlaid with growth data from the same cultures (left axis, diamonds). All growths were repeated in triplicate (*n* = 3) and error bars indicate one standard deviation from the mean value

**Figure 5:**
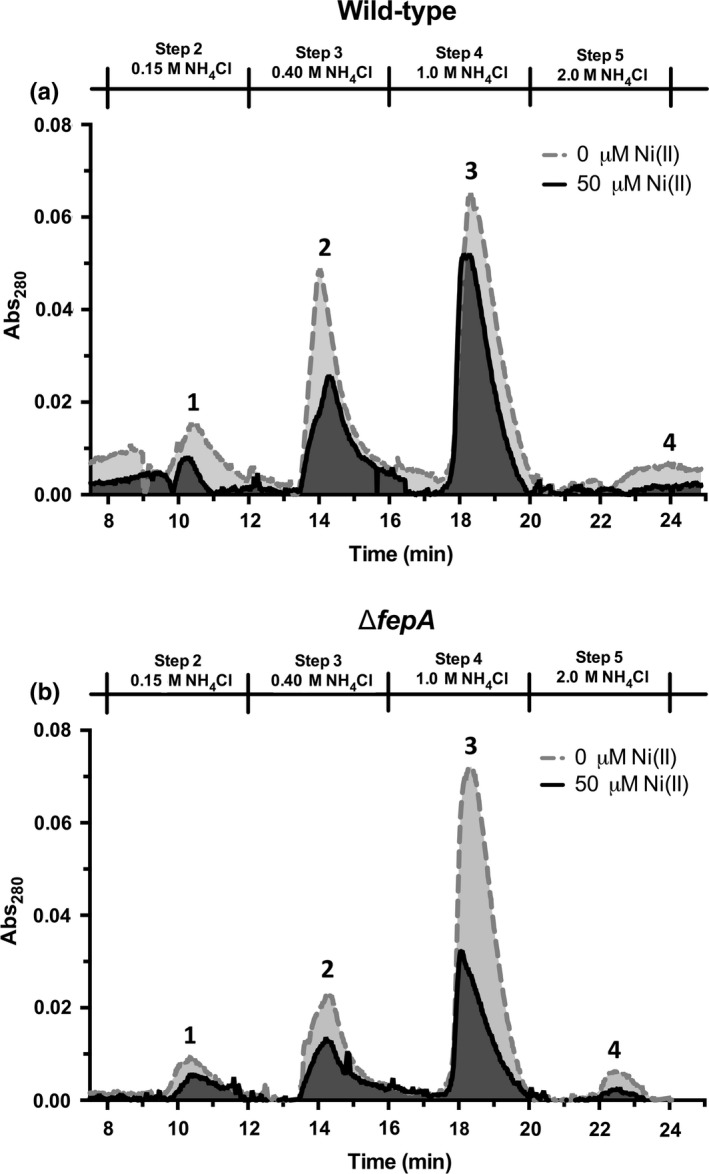
Nickel decreases levels of extracellular enterobactin and its hydrolysis products during lag phase (a) Spent media from Wild‐type MG1655 culture with 0 μM nickel or 50 μM NiCl_2_ was filtered and enterobactin‐related metabolites were separated by FPLC (b) Spent media from Δ*fepA* cultures with no added nickel or 50 μM NiCl_2_ was filtered and enterobactin‐related metabolites were separated by FPLC. Elution times are shown across the bottom axis. The elution peak annotated ‘1’ refers to the linear dimer, ‘2’ refers to the hydrolytically cleaved linear trimer, ‘3’ refers to the nonhydrolytically cleaved linear trimer, and ‘4’ refers to cyclized enterobactin

When iron is limiting, cells export siderophores into the extracellular environment to chelate ferric iron for transport into the cell. The primary siderophore in the strain of *E. coli* used for these studies is the catechol enterobactin. To assess the effects of nickel on siderophore production, catechol levels were monitored in the culture medium using the Arnow assay (Arnow, 1937; Ma & Payne, 2012). Siderophore production is reported in Arnow units, which are normalized for bacterial cell density. Since they are all catechols, this assay should detect enterobactin and its hydrolysis products if they are present in the extracellular medium. The presence of catechols in the culture medium gradually increases over time throughout lag and exponential phase in control cells not exposed to nickel (Figure 4a). In contrast, high‐nickel stress reduces the catechol accumulation in the culture medium (Figure 4a). The decrease in extracellular siderophore levels was proportional to increasing media nickel concentrations (Figure S3). The siderophore enterobactin is imported by FepA after it chelates extracellular ferric iron. To test if the decrease in extracellular siderophore levels during nickel stress was due to an increased rate of clearance of enterobactin by active import via FepA, catechol production also was measured in a *fepA* deletion mutant strain (Δ*fepA*). Siderophore levels were also lower in Δ*fepA* cells exposed to nickel in comparison to untreated cells (Figure 4b). The Arnow assay cannot differentiate enterobactin from its four hydrolysis products, which can also be secreted into the media as low‐affinity iron chelators (Hantke, 1990). To assess the effects of nickel exposure on extracellular enterobactin and its hydrolysis products, fast protein liquid chromatography (FPLC) was used to separate and quantitate enterobactin products from the extracellular medium. In both wild‐type and Δ*fepA* strains the presence of enterobactin and all of its hydrolysis products are lower during nickel exposure (Figure 5a,b). A complementary extraction and analysis protocol using ethyl acetate showed a similar trend in nickel‐dependent reduction in enterobactin and its hydrolysis products during lag phase (Figure S4).

## 4 DISCUSSION

The amount of nickel exposure can proportionally affect the relative growth of *E. coli* cells over time in iron‐limited media. The first stage of growth known as lag phase shows a significant impact from nickel exposure (Figure 1a). The lag phase duration of cells exposed to 50 μM nickel chloride is approximately 10‐fold longer than cells that were not exposed to nickel stress (Figure 1b). According to Rolfe et al., (2012), lag phase is the stage of growth where bacterial cells accumulate iron. Nickel exposure in lag phase results in lower iron accumulation (Figure 2a) and a cellular iron starvation response where genes required for adaptation to iron starvation are constitutively expressed at high levels during lag phase nickel exposure (Figure 2b). Therefore, the disruption of iron homeostasis by lag phase nickel exposure is in good agreement with other recent studies on iron homeostasis and nickel toxicity (Gault et al., 2016; Rolfe et al., 2012). Interestingly, once nickel‐exposed cells exit lag phase, the exponential phase duration (doubling time) shows a much milder twofold increase even at the highest nickel concentration tested. Furthermore, the final cell density reached in stationary phase does not seem to be significantly altered by lag phase nickel exposure (Figure 1a). This result may be partially explained by the selection for a nickel‐resistant mutant population during lag phase, which then grows nearly at wild‐type rates once they accumulate or exit lag phase. In fact, preliminary studies in our laboratory indicate that a nickel‐resistant population is selected for after high‐nickel exposure in lag phase (at concentrations above 30 μM NiCl_2_, data not shown).

Despite the demand for iron and the transcriptional upregulation of iron uptake genes, the level of the siderophore enterobactin and all its hydrolysis products are lower in nickel‐treated cells as compared to untreated control cells. Previously, it was shown that nickel exposure in exponential phase of growth causes repression of iron uptake pathways, including *fepA* and the *entCEBA* operon used for enterobactin synthesis (Gault et al., 2016). The exact mechanism for this inappropriate repression is not clear but may involve a nickel‐dependent increase in intracellular labile iron, perhaps from damaged or mis‐metallated iron proteins, which then triggers inappropriate Fur‐dependent repression of target genes. Using the *lacZ* promoter fusion construct for *fepA*, we also observed similar reduced expression during *exponential* phase nickel exposure (data not shown). However, expression of both *fepA* and *entC* is constitutively high during *lag* phase nickel exposure, as measured by *lacZ* promoter fusions and RT‐qPCR (Figure 3 and S2). Therefore, transcriptional repression of the *entCEBA* system does not explain the observed reduction in enterobactin in the media during lag phase nickel exposure.

Taken together, these findings support the notion that high nickel exposure can disrupt siderophore production in *E. coli* as was previously reported in *Pseudomonas aeruginosa* under iron‐limiting conditions (Visca et al., 1992). These results may provide an additional mechanism to help explain the observed drop in iron accumulation under nickel stress seen in *E. coli* grown under aerobic conditions in minimal media (Gault et al., 2016). Under those growth conditions where ferric iron predominates, the enterobactin uptake pathway would be the main route for iron entry into the cell. However, the results we obtained in lag phase also point to some distinct differences in nickel‐mediated disruption of iron homeostasis between lag and exponential growth phases. In lag phase, the reduction in iron accumulation is much more severe and the Fur and IscR regulons are responding appropriately to the resulting iron starvation. Despite the transcriptional upregulation of the enterobactin synthesis and transport systems, the siderophore is failing to accumulate in the media and is not mediating iron uptake into the cell. The results also indicate that siderophore‐mediated bioremediation may be perturbed if the toxic metal, in this case nickel, also disrupts siderophore production or metabolism.

## CONFLICT OF INTEREST

The authors have no conflicts of interest to declare.

## DATA ACCESSIBILITY STATEMENT

The investigators will share with other researchers, at no more than incremental cost and within a reasonable time, the primary data, samples, physical collections, and other supporting materials created or gathered in the course of this work.
