# Supplementary material for: Nickel exposure reduces enterobactin production in Escherichia coli
Source: Microbiologyopen. 2018 Jul 30;8(4):e00691. doi: 10.1002/mbo3.691 (PMC6460284; doi:10.1002/mbo3.691)
Supplement: Supplementary file 1 [file MBO3-8-e00691-s001.pdf]

Nickel Exposure Reduces Enterobactin Production in *Escherichia coli*

Clorissa L. Washington – Hughes, Geoffrey T. Ford, Alsten D. Jones, Kimberly McRae, F. Wayne Outten*

Department of Chemistry and Biochemistry, University of South Carolina, Columbia, SC, USA.

Running Title: Nickel disrupts siderophore production in *E. coli*

Supplemental Information

Supplemental Experimental Procedures

Total RNA isolation and quantitative RT-PCR

Triplicate cultures of cells were grown for 2 hours in M9 gluconate minimal media with or without 50μM nickel chloride. Cells were harvested by centrifugation and stored at -80°C. RNA was extracted using an acid phenol-chloroform method (Kawano et al., 2002) and stored at -80°C. RNA quality was assessed by UV-visible absorption spectroscopy and gel electrophoresis. 1 μg of total RNA from each RNA sample was used to generate cDNA with the iScript cDNA synthesis kit (BioRad) according to manufacturer's instructions with gene-specific reverse primers added (see below). Levels of cDNA were quantified using qPCR and the SSoAdvanced Universal SYBR Green Supermix (BioRad). Control reactions where reverse transcriptase was omitted showed no signal, indicating there was no contamination of total RNA samples with genomic DNA. ΔCq was calculated by subtracting the average Cq values of triplicate qPCR reactions for the control gene (*ihfB*) from that of the experimental genes (*fepA* or *entC*) for each of the triplicate RNA samples originally isolated. Fold change of mRNA expression is shown as the $2^{-\Delta\Delta Cq}$, where ΔΔCq was calculated by subtraction of the control condition ΔCq (with no nickel added) from the ΔCq of the experimental condition (with nickel added). The following primers were used:

EntC forward primer:5'-CAGGCGATGAAAGAGGTACTG-3',
EntC reverse primer:5'-TCAAAGGGAGTTGCGAGATG-3',
FepA forward primer:5'-CACCTGGTTCCGTAACGATTA-3',
FepA reverse primer:5'-GCACGTTATCCCACTGATAGAG-3',
IhfB forward primer:5'-AGACGGTTGAAGATGCAGTAAA-3',
IhfB reverse primer:5'-GCAAAGAGAAACTGCCGAAAC-3'

Quantification of Enterobactin and its hydroylsis products by LC-MS

Wild-type MG1655 *E. coli* cells were diluted from an overnight culture in 120ml fresh M9 gluconate minimal media and cultured for 2 hours with or without 50µM nickel chloride at 37°C. At 2 hours, culture media was collected after removing cells via centrifugation and was twice extracted using a 1:1 ratio of 100% ethyl acetate to culture medium. The ethyl acetate layer was collected and evaporated by rotovap, resuspended in 10% acetonitrile and 0.1%TFA and analyzed by LC-MS (Winkelmann, 1994). LC-MS samples were analyzed on a Waters QTof API US, quadrupole time-of-flight mass spectrometer and Dionex Ultimate 3000 UPLC. A 30ul injection volume was loaded onto a Chromegabond WR C18, 2.1mmx150mm, 3um particle (ES Industries) column using a binary gradient at a flow rate of 200uL/min. Solvent A: water with 0.1% formic acid and solvent B: acetonitrile with 0.1% formic acid. The run was set to 10%B for 2 minutes, 95% over 20mins, and finally held at 95% for 15mins.

Supplemental References

Kawano, M., Oshima, T., Kasai, H., and Mori, H. (2002) Molecular characterization of long direct repeat (LDR) sequences expressing a stable mRNA encoding for a 35-amino-acid cell-killing peptide and a cis-encoded small antisense RNA in *Escherichia coli*. *Mol. Microbiol.* **45:** 333-49.

Winklemann, G., Cansier, A., Beck, W., and Jung, G. (1994) HPLC separation of enterobactin and linear 2,3-dihydroxybenzoylserine derivatives: a study on mutants of *Escherichia coli* defective in regulation (fur), esterase (fes), and transport (fepA). *Biometals*. **7:** 149-154.

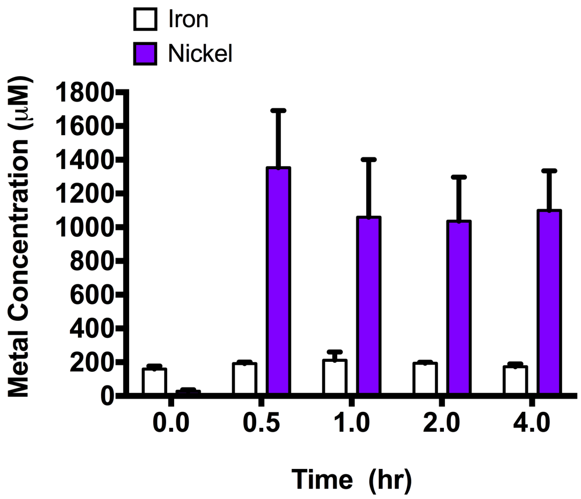

SUPPLEMENTAL FIGURE 1. Intracellular metal concentrations were measured in wild type MG1655 *E. coli* cells that were exposed to 50μM nickel chloride using ICP-MS. All measurements were repeated in triplicate (n = 3) and error bars indicate one standard deviation from the mean value.

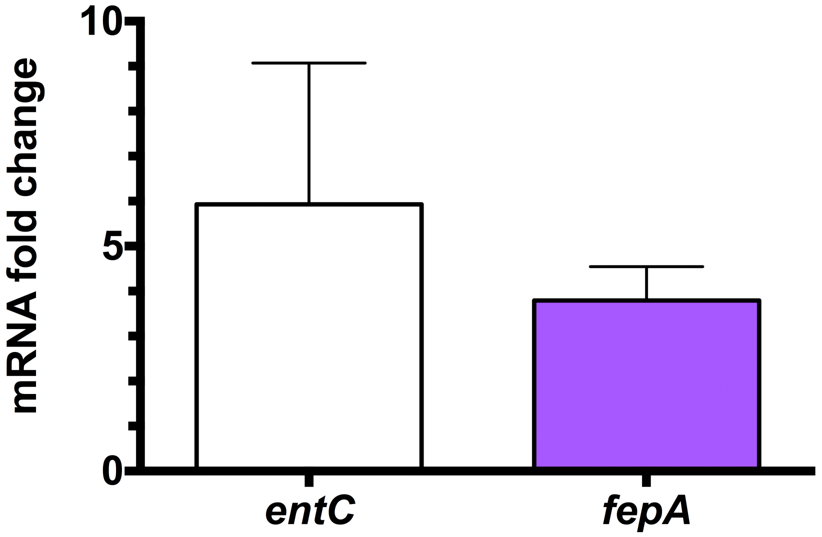

SUPPLEMENTAL FIGURE 2. Fold-induction of *entC* and *fepA* in wild type MG1655 *E. coli* cells that were exposed to 50μM nickel chloride measured using RT-qPCR. Fold change is relative to an untreated culture (see Supplemental Experimental Procedures). All measurements were repeated in triplicate (n = 3) and error bars indicate one standard deviation from the mean value.

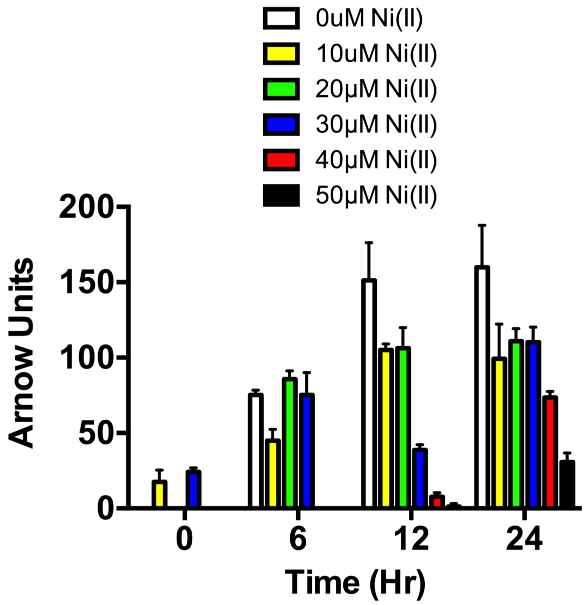

SUPPLEMENTAL FIGURE 3. Total catecholate production in the media is expressed in Arnow units for wild-type MG1655 *E. coli* cells over time with increasing concentrations of nickel. Nickel addition is at time = 0. All growths were repeated in triplicate (n = 3) and error bars indicate one standard deviation from the mean value.

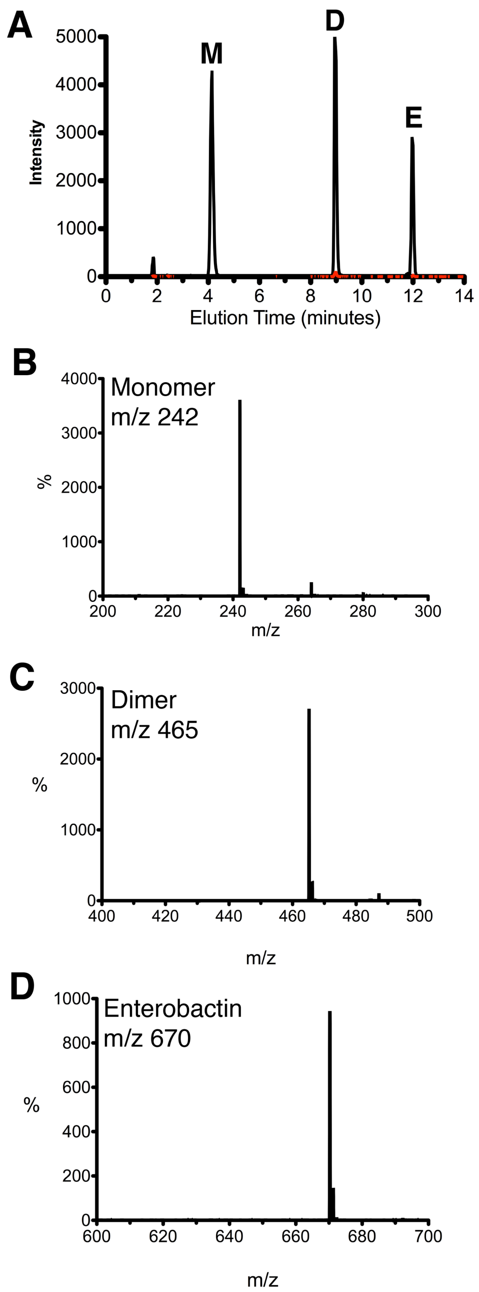

SUPPLEMENTAL FIGURE 4. (A) UPLC separation of enterobactin metabolites from ethyl-acetate extracted spent media from cultures exposed to 0μM (black trace) or 50μM $NiCl_2$ (red trace, nearly undetectable) for 2 hrs in lag phase. Fractions were further analyzed by MS (B-C). See Figure 5 in the main text for further explanation of monomer (M) and dimer (D) hydrolysis products of enterobactin (E).
